# Concentrated Growth Factors (CGF) Induce Osteogenic Differentiation in Human Bone Marrow Stem Cells

**DOI:** 10.3390/biology9110370

**Published:** 2020-10-30

**Authors:** Alessio Rochira, Luisa Siculella, Fabrizio Damiano, Andrea Palermo, Franco Ferrante, Maria Annunziata Carluccio, Nadia Calabriso, Laura Giannotti, Eleonora Stanca

**Affiliations:** 1 Laboratory of Molecular Biology, Department of Biological and Environmental Sciences and Technologies, University of Salento, 73100 Lecce, Italy; alessio.rochira@unisalento.it (A.R.); fabrizio.damiano@unisalento.it (F.D.); nadia.calabriso@ifc.cnr.it (N.C.); laura.giannotti@unisalento.it (L.G.); eleonora.stanca@unisalento.it (E.S.); 2 College of Medicine and Dentistry Birmingham, University of Birmingham, B4 6BN Birmingham, UK; andrea.palermo2004@libero.it; 3 Private Practice, 73100 Lecce, Italy; franco_ferrante@yahoo.it; 4 National Research Council (CNR) Institute of Clinical Physiology (IFC), 73100 Lecce, Italy; maria.carluccio@ifc.cnr.it

**Keywords:** Concentrated Growth Factors (CGF), osteogenic differentiation, hBMSC, bone tissue engineering

## Abstract

**Simple Summary:**

Osteogenesis is a complex physiologic process that occurs during development as well as during damaged bone regeneration. This process requires several growth factors that act on stem cell populations, including Bone Marrow Stem Cells (BMSC). The present study fits into the research field for safe improvement of cell osteogenesis induction. In this context there is a great interest on an autologous and biocompatible blood derived product, named Concentrated Growth Factor (CGF). In particular, the ability of CGF to induce osteogenic differentiation of human BMSC (hBMSC) in vitro was here investigated. The osteogenic differentiation was evaluated measuring typical osteogenic markers such as alkaline phosphatase enzyme activity, matrix mineralization of hBMSC, and expression of some osteogenic-related genes. The results show that CGF alone is able to induce osteogenic differentiation of hBMSC. This finding opens up further, interesting perspectives in the biotechnological use of CGF in the tissue regeneration field.

**Abstract:**

Bone regeneration is a complex process regulated by several factors that control overlapping biological processes, coordinating interactions among distinct cell populations. There is a great interest in identifying new strategies for inducing osteogenesis in a safe and efficient manner. Concentrated Growth Factor (CGF) is an autologous blood derived product obtained by centrifugation of venous blood following the procedure set on the Silfradent device. In this study the effects of CGF on osteogenic differentiation of human Bone Marrow Stem Cells (hBMSC) in vitro have been investigated; hBMSC were cultured with CGF or osteogenic medium, for 21 days. The osteogenic differentiation was evaluated measuring alkaline phosphatase (ALP) enzyme activity, matrix mineralization by alizarin red staining and through mRNA and protein quantification of osteogenic differentiation markers by Real-time PCR and Western blotting, respectively. The treatment with CGF stimulated ALP activity and promoted matrix mineralization compared to control and seems to be more effective than osteogenic medium. Also, hBMSC lost mesenchymal markers and showed other osteogenic features. Our study showed for the first time that CGF alone is able to induce osteogenic differentiation in hBMSC. The application of CGF on hBMSC osteoinduction might offer new clinical and biotechnological strategies in the tissue regeneration field.

## 1. Introduction

Bone regeneration is a complex process regulated by several factors, including pro-inflammatory cytokines, proteins belonging to the Transforming Growth Factor-β (TGF-β) superfamily, and angiogenic factors. These osteoinductive factors are typically embedded within the extracellular matrix, from which they are released during remodeling or trauma. When these proteins are released, they can bind to specific cell surface receptors to initiate signaling cascades, which may ultimately lead to induction of osteogenesis. Remarkably, the biological activities of the osteoinductive factors occur in a spatiotemporally controlled manner, triggering overlapping biological processes, and coordinating interactions among distinct cell populations.

Cell-based therapy for the regeneration of bone tissue has been extensively investigated. Several cell types have been used for the reconstruction of bone tissue, including osteoblasts, embryonic stem cells, periosteum derived-progenitor cells (a specialized cell type that covers bone surfaces and have the potential to differentiate into multiple mesenchymal tissues, including bone) and mesenchymal stem cells, also known as multipotential stromal cells (MSC) [1,2].

MSC have become one of the best alternatives in cell therapy and specifically in bone regeneration. MSC can be isolated virtually from all vascularized tissue and they are able to differentiate into various mesenchymal tissues such as bone, cartilage, muscle, tendon, adipose tissue and hematopoiesis-supporting stroma. However, a growing number of recent reports in the literature have revealed that even if a therapeutic effect can be documented, the osteogenic differentiation of implanted MSC is still not directly demonstrated [3,4].

Bone damages often require surgery with bone graft, a procedure that hides potential and unknown risks for the patients. Therefore, there is a great interest in identifying new and more effective strategies for inducing osteogenesis.

A new autologous biological matrix involved in bone regeneration in vivo [3,4,5] and osteoblast differentiation [6,7] is Concentrated Growth Factor (CGF). CGF is a fibrin biomaterial rich in growth factors obtained by centrifugation of venous blood, at alternating speeds, as set on the Silfradent device [8]. This is the third-generation of platelet rich product. In contrast to platelet-rich plasma (PRP), that requires addition of thrombin and anticoagulant and a double centrifugation technique for preparation [9], CGF requires a simple preparation, since manipulation of the product is necessary when exclusively autologous blood product is used without the addition of other substances [10]. The centrifugation protocol developed by Sacco produced a fibrin clot stiffer than platelet rich fibrin (PRF) [11]. The differences in the preparation method between CGF and PRF are the basis of the structural and functional characteristics of these similar but distinct preparations. PRF has shown enhancing effects on stem cell proliferation, differentiation, migration, and mineralization during bone formation [9,12,13]; however, PRF alone may have an unstable effect on osteogenesis in vivo [13]. CGF releases TGF-β, Vascular Endothelial Growth Factor (VEGF), Platelet-Derived Growth Factor (PDGF), Bone Morphogenetic Protein 2 (BMP2) and Insulin-like Growth Factor (IGF-1) [14,15,16] that increase proliferation and extracellular matrix mineralization via the BMP2/SMAD5/RUNX2 signaling pathway [6]. CGF also contains CD34 positive cells, fibroblasts, leukocyte and endothelial cells for angiogenesis and tissue remodeling and provides a matrix for cell migration [8,14]. Furthermore, several studies show the osteogenic effect of CGF in vivo [3,4,5,9] and in vitro [5,6,7].

Human skeletal stem cells, also known as human Bone Marrow Stromal Cells (hBMSC), are non-hematopoietic stromal cells that reside in a perivascular niche within the bone marrow stroma [17]. They are defined in vitro as plastic-adherent cells expressing a constellation of surface markers CD 90, CD 73 and CD 105 and having the capacity for multi-lineage differentiation into osteoblast, adipocyte and chondrocyte [18]. The “stemness” characteristic phenotype of BMSC is based on their ability to form heterotopic bone and bone marrow organ during serial transplantation in vivo, a characteristic exhibited only by a fraction of BMSC [19,20]. Several studies have clearly demonstrated that BMSCs have great potency for promoting the regeneration of bone defects in animal models due to their high capacity for self-renewal and multipotentiality for differentiation [3,4,5,21,22].

The aim of this work was to investigate the effect of CGF in promoting the osteogenic differentiation of hBMSC in vitro. Our study showed that CGF stimulated alkaline phosphatase (ALP) activity and promoted matrix mineralization in hBMSC. Furthermore, after incubation with CGF, hBMSC lost mesenchymal markers and showed other osteogenic features. This experimental model could represent a simplified pattern for future applications in the field of regenerative medicine. Therefore, CGF might be a very promising material for bone regeneration. The application of CGF on hBMSC osteoinduction might offer new clinical and biotechnological strategies in the human tissue regeneration field.

## 2. Results

### 2.1. CGF Increased ALP Activity in hBMSC

**Figure 1:**
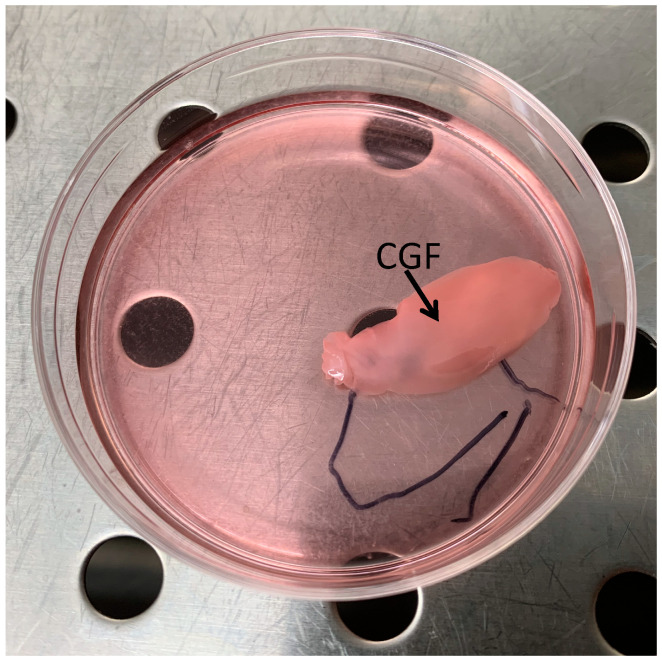
Monostrate of human Bone Marrow Stem Cells (hBMSC) in the presence of Concentrated growth factors (CGF). Immediately after its preparation CGF was placed directly on monolayer hBMSC culture, in Mesenchymal Stem Cell (MSC) Basal medium, for the indicated times.

**Figure 2:**
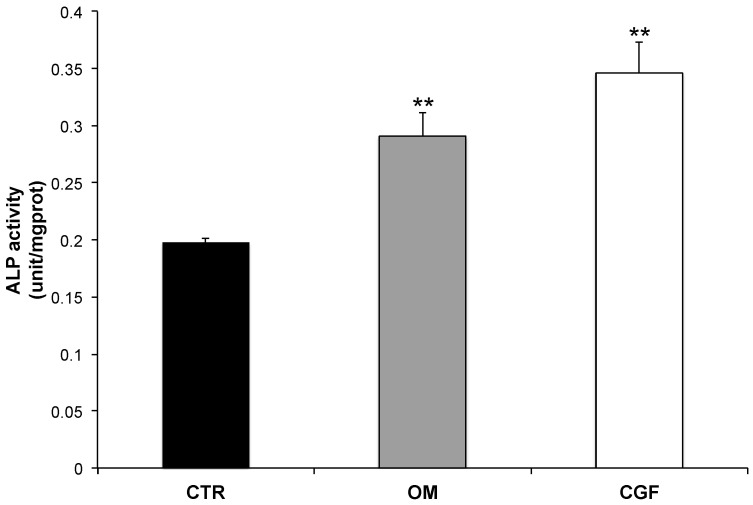
Alkaline phosphatase (ALP) activity upon osteogenic differentiation. Enzymatic activity was detected in hBMSC cultured in MSC Basal Medium (BM) (Control, CTR), Osteogenic Medium (OM), BM + CGF (CGF), for 14 days. The results were expressed as the means ± SD of duplicate measurements from three independent experiments (** *p* < 0.01 versus CTR).

To study the effect of CGF on osteogenic differentiation, hBMSC were incubated with CGF for 14 days to perform ALP activity assay or 21 days to carry out all the other experiments (Figure 1). The effect of CGF on osteogenic differentiation of hBMSC was first investigated by measuring ALP enzyme activity. The enzymatic assay was carried out after 14 days of cell incubation in Basal medium + CGF or in Osteogenic medium (OM), since ALP is an early osteogenic marker. In untreated control cells, (CTR, hBMSC cultured in BM) the enzyme activity of ALP was about 0.2 units per mg of proteins (Figure 2). In hBMSC cultured in OM, used as a positive control, ALP enzymatic activity enhanced by about 50% with respect to CTR (Figure 2), whereas CGF treatment induced an increment of ALP activity higher than 70% with respect to CTR (*p* < 0.01) reaching values of about 0.35 units per mg of proteins (Figure 2).

### 2.2. Effect of CGF on Matrix Mineralization and Surface Markers Expression

**Figure 3:**
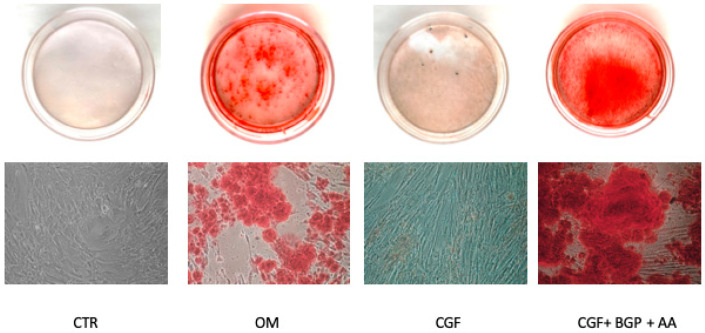
Alizarin Red staining in hBMSC cultured in MSC Basal Medium (BM) (Control, CTR), Osteogenic Medium (OM), BM + CGF (CGF), or BM + CGF + β-Glycerophosphate (BGP)+ Ascorbic Acid (AA) (CGF + BGP + AA) for 21 days (20x).

To further assess the effects of CGF on osteogenic differentiation, the matrix mineralization of hBMSC was evaluated by Alizarin red staining (ARS) experiments. After 21 days the staining was not evident in untreated control hBMSC whereas it was significantly revealed in positive control (OM-treated cells). The treatment with CGF induced cell morphology modifications in hBMSC, although a very low red staining was detected (Figure 3). We performed ARS staining up to 28 days of CGF treatment, obtaining similar data compared to 21 days of CGF treatment (Supplementary Figure S1), suggesting that a longer differentiation time did not modify ARS staining in CGF-treated hBMSC. As widely reported [23,24,25,26], in hBMSC the matrix mineralization requires the addition of substrates such as β-glycerophosphate (BGP) and ascorbic acid 2-phosphate (AA); in fact, CGF plus BGP and AA strongly increased ARS, demonstrating a positive effect on matrix mineralization (Figure 3).

**Figure 4:**
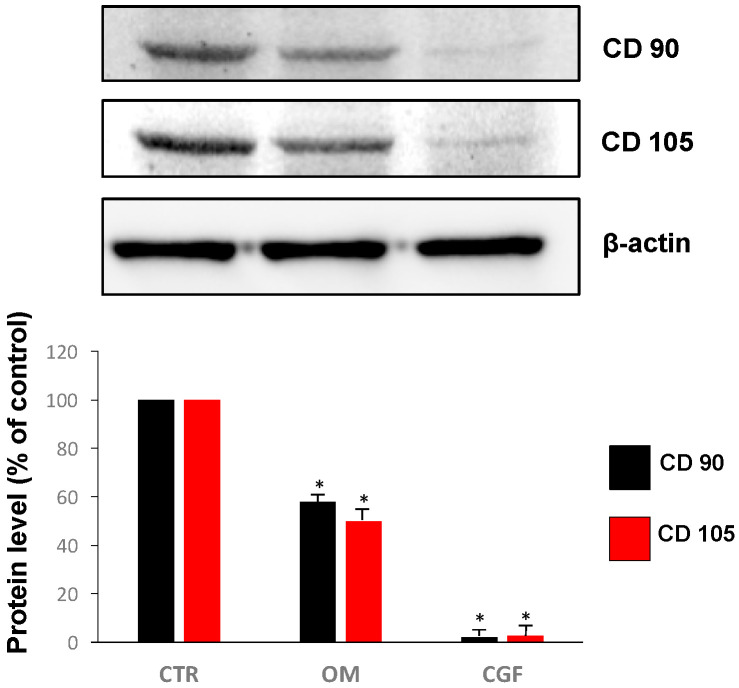
Expression of surface proteins CD 90 and CD 105 in hBMSC cultured in MSC Basal Medium (BM) (Control, CTR), Osteogenic Medium (OM) or BM + CGF (CGF) for 21 days. The results were expressed as the means ± SD of duplicate measurements from three independent experiments (* *p* < 0.05 versus CTR).

Stem cells are known to lose the expression of their specific surface markers when they differentiate; among these specific markers, the decrease of CD 90 and CD 105 expression has been reported as differentiation signal [27]. In order to clarify whether CGF could determine hBMSC differentiation, Western blotting quantification of CD 90 and CD 105 protein contents was carried out. As shown in Figure 4, CGF abolished the expression of both CD 90 and CD 105 proteins. Note that OM treatment, used as a positive control, only reduced CD 90 and CD 105 by about 40% and 50%, respectively.

### 2.3. CGF Increased the Expression of Osteogenic Differentiation Markers

**Figure 5:**
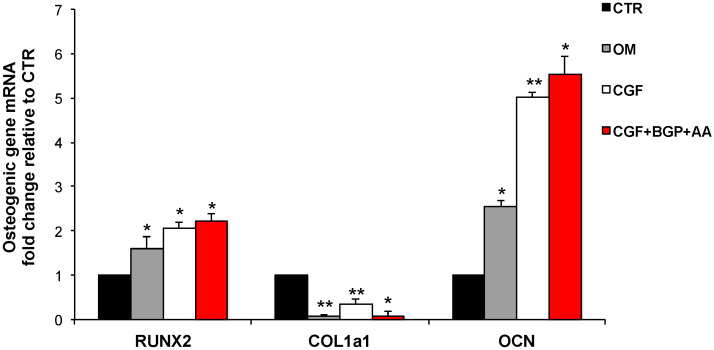
Effect of CGF on osteogenic gene expression. mRNA abundance of RUNX2, COL1a1, OCN in hBMSC cultured in MSC Basal Medium (BM) (Control, CTR), Osteogenic Medium (OM), BM + CGF (CGF), or BM + CGF + β-Glycerophosphate (BGP) + Ascorbic Acid (AA) (CGF+BGP+AA) for 21 days. *Gapdh* was used as housekeeping gene for normalization. Fold change in mRNA expression was relative to CTR. The results were expressed as the means ± SD of triplicate measurements from three independent experiments (* *p* < 0.05; ** *p* < 0.01 versus CTR).

To evaluate the effects of CGF on hBMSC osteogenic differentiation, the mRNA abundance of RUNX2, the transcription factor key regulator of osteogenesis, of COL1a1 and of OCN, extracellular matrix proteins used as osteogenic differentiation markers, was quantified (Figure 5). The treatment with CGF and CGF + BGP + AA markedly augmented the mRNA abundance of RUNX2 and OCN with respect to CTR. Indeed, the expression of RUNX2 increased by about 2 and 2.2 fold, in CGF- and CGF + BGP + AA-treated hBMSC, respectively, whereas OCN mRNA levels augmented by about 5 and 5.5 fold in CGF- and CGF + BGP + AA-treated hBMSC, respectively, when compared to CTR. RUNX2 and OCN mRNA levels, significantly increased in cells incubated in OM as well, with respect to CTR, by about 1.6 and 2.5 fold, respectively, as expected. COL1a1 expression behaves differently: all the treatments induced a statistically significant reduction of COL1a1 mRNA abundance, when compared to CTR (Figure 5).

Since similar values in gene expression of osteogenic markers, RUNX2 and OCN, were detected in CGF- and CGF+BGP+AA-treatment, therefore, the following analysis will be performed with CGF only.

**Figure 6:**
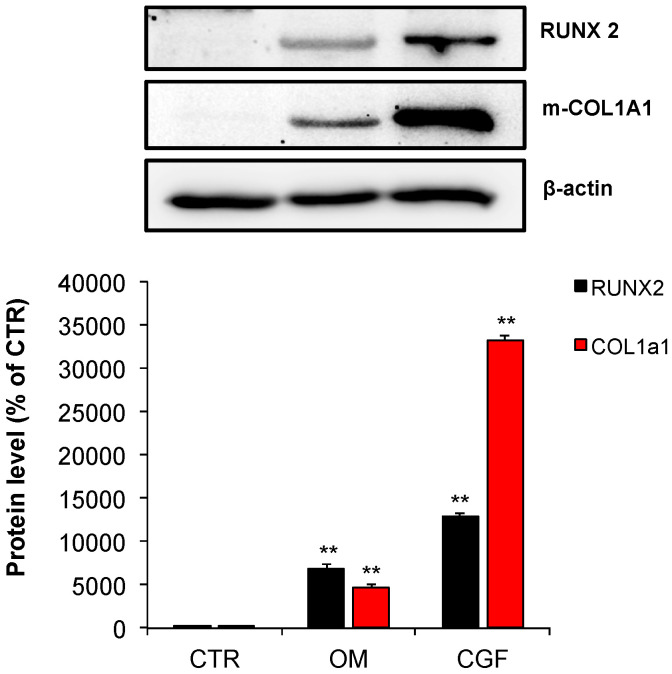
Expression of osteogenic proteins in hBMSC cultured in MSC Basal Medium (BM) (Control, CTR), Osteogenic Medium (OM) or BM + CGF (CGF) for 21 days. m-COL1a1: mature COL1a1. The results were expressed as the means ± SD of duplicate measurements from three independent experiments (** *p* < 0.01 versus CTR).

Next, experiments were carried out to investigate the CGF effect on the proteins involved in osteogenic differentiation of hBMSC. Therefore, the protein content of RUNX2 and COL1a1 was quantified. The obtained results show that, in hBMSC, CGF determined a very strong increase of both RUNX2 (130-fold) and mature COL1a1 (mCOL1a1) (330-fold) protein levels when compared to the untreated CTR cells. OM increased both RUNX2 (70-fold) and mCOL1a1 (50-fold), as well, when compared to CTR. Interestingly, the increment induced by the CGF treatment was remarkably higher than that caused by the OM on the same protein (Figure 6).

### 2.4. Localization of RUNX2

**Figure 7:**
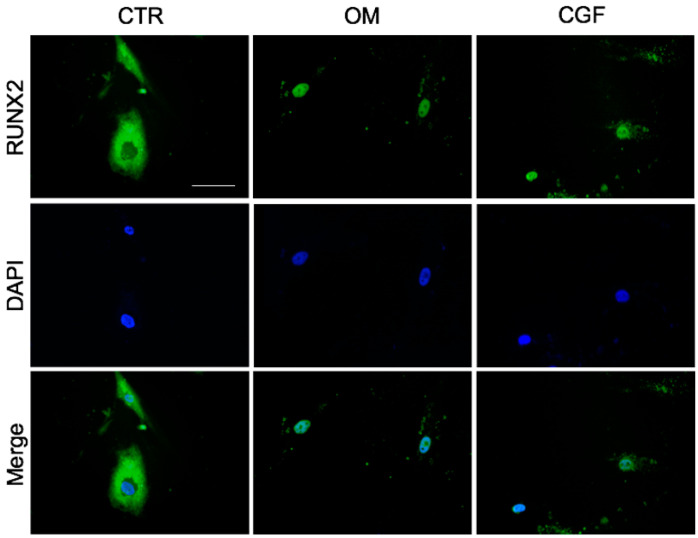
Nuclear translocation of RUNX2 during osteogenic differentiation. Immunofluorescent staining for RUNX2 in hBMSC cultured in MSC Basal Medium (Control, CTR), Osteogenic Medium (OM) or BM + CGF (CGF) for 21 days (40X), performed by using mouse monoclonal antibody against RUNX2. Green, goat anti-mouse immunoglobulins conjugated with AlexaFluor 488; blue, nuclear staining with DAPI. Scale bar: 50 μm.

RUNX2 is a transcriptional factor required for bone and tooth development. It modulates the formation of the regulatory complex involved in transcription activation or repression of specific genes during osteogenic differentiation. The subcellular localization of RUNX2 protein was assessed by immunocytochemistry. Figure 7 shows that RUNX2 was expressed in the cytosol of hBMSC grown for 21 days in BM, whereas in the cells cultured in OM it was localized in the nucleus. In hBMSC grown in presence of CGF, RUNX2 protein was also localized in the nucleus (Figure 7).

## 3. Discussion

Bone defect reconstruction and trauma repair are of crucial importance in orthopedic surgery and odontostomatology fields. There is growing attention on a combined CGF with BMSC approach for in vivo bone regeneration [3,4].

CGF, the third-generation platelet concentrate, derives from modification of preparation protocol of PRP and PRF. The osteogenic effects of these fractions have been extensively studied [28,29,30] but the ability of PRP and PRF to induce in vivo osteogenic differentiation is still debated [12,29]. On the other hand, several studies showed that CGF is able to improve bone regeneration in vivo [3,4,5].

Although in previous studies BMSC cells were used in combination with CGF in animal osteogenesis models in vivo [3,4], no molecular data have been available so far, demonstrating CGF-induced osteogenic differentiation of hBMSC. Here, we reported that CGF exerts a remarkable osteoinductive effect on hBMSC after 21 days of treatment. These results have been obtained by placing CGF in contact with the hBMSC directly in the culture plate (Figure 1).

In all the experiments the osteogenic differentiation of hBMSC was assessed also in hBMSC cultured in OM, which represented the positive control.

To evaluate the osteogenic differentiation efficiency induced by CGF the ALP activity was detected. ALP is considered an early osteogenic marker [31,32], therefore ALP activity has been analyzed after 14 days of treatment. Our results showed that CGF increased ALP activity with respect to CTR. The increment was even higher than that observed in OM-treated hBMSC (Figure 2). All the other experiments were carried out after 21 days.

OM caused the formation of mineralized nodules in hBMSC in vitro after 21 days, revealed by ARS, as expected. Although CGF alone, in basal medium, determined only morphological changes in hBMSC shape without a consistent matrix deposition, the addition of the mineral precursors, BGP and AA, was sufficient to provide the complete mineralization of hBMSC. Note that hBMSC mineralization induced by CGF+BGP+AA was stronger than that caused by OM (Figure 3).

Is widely known that stem cells express specific surface antigens, and the lack of their expression is characteristically considered a differentiation signal [27]. Our study showed that CGF treatment determined the complete differentiation of hBMSC cells since the expression of stem cell surface markers CD 90 and CD 105 dramatically decreased; furthermore the CD 90 and CD 105 reduction induced by CGF was much greater than that caused by OM, both compared to CTR (Figure 4).

In order to verify the hBMSC osteogenic differentiation induced by CGF, the expression of some molecular targets typically activated during osteogenic differentiation has been investigated. The data on the molecular mechanisms of osteogenic induction of human mesenchimal stem cells (hMSC) are various and in some cases contrasting. RUNX2 represents the master regulator transcription factor in osteogenesis [33,34]. It has been reported that in hBMSC the glucocorticoid dexamethasone induces osteogenesis through the activation of RUNX2, without modifying its expression [35]. On the other hand, other findings reveal that osteogenic differentiation of hMSC induced by dexamethasone requires the activation of RUNX2 expression [23,36,37]. Our findings demonstrate that CGF treatment strongly increased RUNX2 expression, at mRNA and protein levels. This effect was more pronounced than that caused by OM when compared to CTR (Figure 5 and Figure 6). It has been reported that the nuclear or cytoplasmic localization of RUNX2 is associated with its transcriptional activity; precisely the nuclear localization of RUNX2 is functionally linked with its biological activity as a bone-related transcriptional factor [38]. As shown in Figure 7 both CGF and OM clearly determined RUNX2 nuclear translocation, whereas the protein was localized into the cytosol of undifferentiated hBMSC (CTR). COL1a1, a protein commonly considered a marker of the activated osteogenic differentiation of cells, is produced as precursor protein and then undergoes post-translational modifications to give mature protein [39]. Therefore, the mature form of COL1a1 (mCOL1a1) was quantified by Western blotting. Likewise to RUNX2, in CGF-treated hBMSC, an increment of mCOL1a1 protein level, was found (Figure 6). The mRNA abundance of COL1a1 was reduced upon CGF treatment with respect to CTR, since COL1a1 expression increases at the beginning of osteoblastic differentiation as well as ALP activity [33], whereas the COL1a1 mRNA was quantified at the end of cell differentiation process (Figure 5). Instead, a sharp increase of OCN mRNA abundance in CGF-treated hBMSC was found with respect to CTR, after 21 days, and this increment was even greater than that observed in OM-treated hBMSC with respect to CTR (Figure 5). In fact, OCN is used as a marker of last-stage osteogenic differentiation [33].

Note that similar values in gene expression of osteogenic markers, RUNX2 and OCN, were detected in CGF- and CGF+BGP+AA-treatment (Figure 5) suggesting that osteogenic differentiation in the presence of CGF did not require the presence of BGP and AA. The addition of BGP and AA is necessary only to detect matrix mineralization by alizarin red staining (Figure 3). Therefore, we did not include the CGF+BGP+AA sample in Western blotting, IF and ALP activity experiments. The osteogenic effect exerted by CGF is remarkable as shown by our data. This finding leads us to speculate on the existence of different molecular mechanisms of hBMSC osteogenic induction by CGF, promoted by different components of CGF, that may act synergistically. The complex nature of CGF lies in the presence, in addition to the fibrin, of various growth factors and a heterogeneous cellular component [8,13]. Our results can be explained by the presence in CGF of multiple growth factors that regulate cell differentiation through the activation of several signaling pathways.

Among the growth factors identified in the CGF, TGF- β and BMP-2 promote the expression of RUNX2 through the activation of the respective cell signaling pathways [40]. Wnt/β-catenin signaling pathway has a potent ability to induce osteogenesis. Following activation, β-catenin ultimately promotes the expression of RUNX2 through the activation of TCF/LEF transcription factors [41]. However, whether CGF components could activate the Wnt/β-catenin signaling pathway remains to be investigated.

Furthermore, a previous study has shown that CGF promoted the proliferation, osteogenic maturation and mineralization of rat mesenchymal stem cells, dramatically increased the expression levels of RUNX2 and OCN, and implied a role of BMP-2/Smad signaling pathway in promoting the osteogenic differentiation of BMSC [42].

It has also been reported that CGF stimulated the proliferation and osteogenic differentiation of gingiva-derived mesenchymal stem cells by regulating the expression of BMP2 and RUNX2 [43]. CGF significantly increased the ALP activity and upregulated the expression and secretion of osteogenic differentiation markers, including COL1 and OCN, in rabbit periosteum-derived cells [44].

Moreover, Yu et al., showed that CGF induced periodontal ligament stem cell differentiation into osteoblasts in vitro and sped up osteogenesis during the differentiation process [45]. Recently, in vitro and in vivo studies on bone regeneration, reporting the use of adipose-derived stem cells (ADSC), are receiving considerable attention, due to the high proliferative capacity and osteogenic differentiation potential of ADSC. Ma et al., showed that CGF promoted osteogenic proliferation and differentiation in beagle ADSC by stimulating ALP activity and the expression of genes associated with osteogenesis, such as COL1 and RUNX2 [46]. Furthermore, multiple growth factors concentrated in PRP were shown to be effective in inducing proliferation and osteogenic differentiation of ADSC [47]. Taken together, our results reveal that CGF could represent an excellent biomaterial in bone tissue engineering, having a beneficial impact on osteogenic differentiation of human BMSC, and this effect appears to be stronger than that caused by OM, used as a positive control of the process.

These findings related to the osteogenic properties of CGF highlight its potential in clinical applications, including oral and maxillofacial surgery. It has been reported that CGF effects improved the regeneration of hard and soft tissues in prosthetic, post traumatic or post-surgical rehabilitation and in implantology. In fact, CGF can be used as a filling material in bone defect repair to avoid the use of xenografts [48,49,50,51].

However, some limitations are involved in the present study. First, we evaluated the pro-osteogenic properties of the whole CGF, which may be related to multiple growth factors. In fact we did not manipulate CGF to avoid alterations in its structure and composition. However, the release kinetics of unaltered and unmodified CGF remain to be clarified. From the perspective of clinical research, it is important to characterize the whole CGF in order to better evaluate its potential in tissue regeneration. To date, there are some studies that analyzed the release of growth factors only by manipulated CGF [11,16]. Therefore, this important aspect needs to be investigated in depth in a future work.

Moreover, our study was conducted in a preclinical model represented by cultured hBMSC, known as a reliable, challenging tool for in vitro osteogenesis. Nevertheless, experimental findings in vitro do not necessarily translate directly to the situation in vivo. Therefore, further investigations, including human trials, are necessary to evaluate the in vivo effectiveness of CGF in improving osteogenesis.

## 4. Conclusions

This work demonstrated that CGF, exerting a direct effect on the osteogenic differentiation of hBMSC, could represent a very promising material for bone regeneration, opening new interesting perspectives in the use of CGF in the tissue regeneration field.

## 5. Materials and Methods

### 5.1. Cell Culture, Reagents and Antibodies

Human Bone Marrow-Derived Mesenchymal Stem Cells, hBMSC (ATCC-PCS-500-012), Mesenchymal Stem Cell Basal Medium (ATCC PCS500030) and Mesenchymal Stem Cell Growth Kit for Bone Marrow-derived MSC containing FGF-b and IGF-1 (ATCC PCS500041) were purchased from ATCC (Milan, Italy). BMSC from three different lots were used to perform all experiments. Cells were used between the third and the sixth passages. DMEM, Fetal bovine serum (FBS), penicillin/streptomycin, L-glutamine were purchased from Corning (Manassas, VA, USA); BGP, dexamethasone, AA, were obtained from Sigma Chemical Co. (Milan, Italy).

CD 90, CD 105, RUNX2, COL1A1, goat anti-mouse IgG conjugated with peroxidase, and β-actin antibodies were purchased from Santa Cruz Biotechnology, (Santa Cruz, CA, USA).

Goat anti-mouse conjugated with AlexaFluor 488 was purchased from Bethyl Laboratories Inc. (Montgomery, TX, USA).

### 5.2. Preparation of CGF

Blood samples of 8 mL were taken via venipuncture from five donors who were non-smokers and in good general health. For each set of experiments CGF was prepared from the same blood sample of a single donor. Tubes of blood were processed by a device (Medifuge MF200; Silfradent srl, Forlì, Italy) to obtain CGF, following the manufacturer’s instructions [10]. In all the experiments with CGF-treated hBMSC, CGF, as it is, was placed directly into the cell dishes for the reported times (Figure 1). Informed consents were obtained from the donors included in this study.

### 5.3. Osteogenic Differentiation Protocol

Undifferentiated hBMSC were cultured in Mesenchymal Stem Cell Basal Medium (BM) supplemented with 7% FBS, 100 IU/mL penicillin/streptomycin, 2.4 mM, 125 pg/mL FGF-b and 15 ng/mL IGF-1, at a density of 5 × 10^3^ cells/cm^2^ and incubated for 24 h at 37 °C under 5% CO_2_. To induce osteogenic differentiation hBMSC were cultured in OM, (DMEM with 10% FBS, 100 IU/mL penicillin/streptomycin, 2 mM L-glutamine, 10 mM β-glycerophosphate, 100 nM dexamethasone, 100 μM ascorbic acid 2-phosphate), or cultured in BM with CGF supplemented with 7% FBS, 100 IU/mL penicillin/streptomycin (CGF) (Figure 1), or with CGF supplemented with 7% FBS, 100 IU/mL penicillin/streptomycin, 10 mM β-glycerophosphate (BGP), 100 μM ascorbic acid 2-phosphate (AA) (CGF + BGP + AA) for 21 days. The medium was replaced at a rate of 50% every 3 days.

### 5.4. ALP Activity Assay

The hBMSC were cultured with BM, OM, or CGF conditioned medium for 14 days. The cultured medium was refreshed every three days. ALP activity was measured by using an ALP assay kit (MyBioSource) according to the manufacturer’s instructions. Cells were washed twice with PBS and collected by cell scraper in cold PBS. The enzymatic activity of ALP was monitored through the conversion of 4-aminopyrline in red quinone derivative. The absorbances were detected at OD = 520 nm. The activity was normalized with respect to the total proteins and the values of enzyme activity were expressed in Unit/mg proteins. One ALP activity unit is defined as the amount of 1 mg phenol produced by 1 mg proteins that react with the substrate in 15 min.

### 5.5. Alizarin Red Staining

Alizarin red S stain (Sigma) solution was prepared as described in [16]. Briefly, Alizarin red S stain 2% solution in distilled water was adjusted to pH 4.2 by adding ammonium hydroxide drop by drop while stirring, using an electrode pH meter. The solution was then filtered through a 0.45 μm microfilter (Millipore Corporation, Bedford, MA, USA) and kept in an amber bottle. This solution was refiltered through a 0.22 μm microfilter immediately before use. The hBMSC, 4.5 × 10^4^ viable cells/mL, were seeded in 35 mm ∅ culture dishes. After 24 h, the culture medium was refreshed. Cells were grown in BM, OM or BM with CGF, for 21 days. ARS of hBMSC cells was performed at 21 days to detect osteoblast calcification. Cells were washed twice with PBS, fixed in 4% (*v/v*) paraformaldehyde in PBS for 15 min, washed with distilled water three times, and then stained by Alizarin Red S staining solution. After being rinsed twice with distilled water, the cells were photographed.

### 5.6. Real-Time PCR

Total RNA was extracted from cells grown in a 60 mm ∅ culture dish using the GenEluteTM Mammalian total RNA miniprep kit (Sigma, Merck Life Science S.r.l., Milan, Italy) following the manufacturer’s protocol. The reverse transcriptase reaction (20 μL) was carried out using 1 μg of total RNA, random primers and MultiScribe^®^ Reverse Transcriptase (Applied Biosystem) according to the manufacturer’s protocol. Quantitative gene expression analysis was performed in a CFX Connect Real time System (Biorad) using SYBR Green technology (FluoCycle-Euroclone, Milan, Italy). Primers used in Real time PCR were reported in Table 1. The efficiency of each primer was tested running a standard curve in duplicate. The quantifications were performed using the ΔΔCT method and Gapdh gene was used as an internal control for normalization. Fold change in mRNA expression was relative to CTR. The specificity of PCR products was confirmed by melting curve analysis. The identity of the amplified products was confirmed by sequencing analysis.

### 5.7. Western-Blot Analysis

To obtain whole protein cell extracts for Western-blot analysis, cells were scraped in the following buffer: 20 mM Tris–HCl (pH 8.0), 420 mM NaCl, 2 mM EDTA, 2 mMNa3VO4, and 1% (*v/v*) Nonidet P-40, supplemented with a cocktail of protease inhibitors. Cells were then passed several times through a 20-gauge syringe and centrifuged at 16,000× g for 20 min at 4 °C. Proteins in homogenate were determined using the Bio-Rad protein assay kit. Lyophilized bovine serum albumin (BSA) was used as a standard. Total cell proteins were dissolved in sodium dodecyl sulphate (SDS) sample buffer and separated on 10% (*w/v*) SDS gels. Separated proteins were then transferred electrophoretically onto a nitrocellulose membrane (Pall, East Hills, NY, USA). Equal protein loading was confirmed by Ponceau S staining. The filter was blocked with 5% (*w/v*) non-fat dried milk in buffered saline. Blots were incubated with specific primary antibodies and the immune complexes were detected using appropriate peroxidase-conjugated secondary antibodies and enhanced chemiluminescent detection reagent (Amersham International, Little Chalfont, UK) [52]. Densitometric analysis was carried out on the Western-blots by using the ChemiDoc MP Image System (BioRad, Hercules, CA, USA).

### 5.8. Immunofluorescence Localization of RUNX2

hBMSC, 4.5 × 10^4^ viable cells/mL, were seeded on sterile glass cover slips. After 24 h, the culture medium was refreshed. Cells were grown in BM, or in OM or in BM with CGF for 21 days. The cells were briefly rinsed three times with sterile PBS, at 4 °C, before fixing in 4% (*v/v*) paraformaldehyde in PBS for 15 min. Cell samples were washed three times with PBS, permeabilized with PBS pH 7.4 containing 0.1% (*v/v*) Triton X-100 and 1% (*w/v*) BSA for 30 min and then incubated with 2.5% BSA in PBS for further 30 min to block nonspecific binding sites.

Cells were incubated with mouse monoclonal antibody against RUNX2 diluted 1:250 with 2.5% (*w/v*) BSA in PBS for 1 h at RT, then cells were washed three times with PBS and incubated with goat anti-mouse immunoglobulins conjugated with AlexaFluor 488 (Bethyl Laboratories Inc., Montgomery, TX, USA) for 1 h in the dark. Appropriate control with hBMSC cells incubated without the first antibody was performed.

Immunocomplexes were visualized with Invitrogen^TM^ EVOSTM FL auto digital inverted fluorescence system.

### 5.9. Statistical Analysis

Values were expressed as mean ± SD for the indicated number of experiments. Differences between two groups were settled by unpaired Student’s t tests. In all comparisons, *p* < 0.05 was considered as statistically significant.

## Figures and Tables

**Table 1:** Oligonucleotides used for Real-time PCR analysis.

Gene Name	Acession Number	Sequences	pb
*RunX2*	NM_001278478.2	F: gacaaccgcaccatggtgg R: tctggtacctctccgaggg	160
*Col1a1*	NM_000088.3	F: agggaatgcctggtgaacg R: gagagccatcagcacctttg	90
*Ocn*	NM_199173.6	F: gctacctgtatcaatggct R: cgatgtggtcagccaactc	111
*Gapdh*	AJ005371.1	F: atggccttccgtgtccccac R: acgcctgcttcaccaccttc	245

*RunX2*, runt-related transcription factor 2; *Col1a1*, collagen type I α1; *Ocn*, Osteocalcin; *Gapdh*, glyceraldehyde-3-phosphate dehydrogenase.

## Supplementary Materials

The following are available online at https://www.mdpi.com/2079-7737/9/11/370/s1, Figure S1: Alizarin Red staining in hBMSC cultured for 28 days.

## References

[1] Ho-Shui-Ling A., Bolander J., Rustom L.E., Johnson A.W., Luyten F., Picart C. (2018). Bone regeneration strategies: Engineered scaffolds, bioactive molecules and stem cells current stage and future perspectives. Biomaterials.

[2] Ansari M. (2019). Bone tissue regeneration: Biology, strategies and interface studies. Prog. Biomater..

[3] Honda H., Tamai N., Naka N., Yoshikawa H., Myoui A. (2013). Bone tissue engineering with bone marrow-derived stromal cells integrated with concentrated growth factor in Rattus norvegicus calvaria defect model. J. Artif. Organs.

[4] Chen X., Wang J., Yu L., Zhou J., Zheng D., Zhang B. (2018). Effect of Concentrated Growth Factor (CGF) on the promotion of osteogenesis in Bone Marrow Stromal Cells (BMSC) in vivo. Sci. Rep..

[5] Takeda Y., Katsutoshi K., Matsuzaka K., Inoue T. (2015). The effect of concentrated growth factor on rat bone marrow cells in vitro and on calvarial bone healing in vivo. Int. J. Oral Maxillofac. Implant..

[6] Tian S., Wang J., Dong F., Du N., Li W., Song P., Liu Y. (2019). Concentrated growth factor promotes dental pulp cells proliferation and mineralization and facilitates recovery of dental pulp tissue. Med. Sci. Monit..

[7] Xu F., Qiao L., Zhao Y., Chen W., Hong S., Pan J., Jiang B. (2019). The potential application of concentrated growth factor in pulp regeneration: An in vitro and in vivo study. Stem Cell Res. Ther..

[8] Bernardi S., Mummolo S., Tecco S., Continenza M.A., Marzo G. (2017). Histological characterization of Sacco’s concentrated growth factors membrane. Int. J. Morphol..

[9] Kim T.-H., Kim S.-H., Sándor G.K., Kim Y.-D. (2014). Comparison of platelet-rich plasma (PRP), platelet-rich fibrin (PRF), and concentrated growth factor (CGF) in rabbit-skull defect healing. Arch. Oral Biol..

[10] Sacco L. (2006). Lecture, International Academy of implant prosthesis and osteoconnection. Lecture.

[11] Masuki H., Okudera T., Watanebe T., Suzuki M., Nishiyama K., Okudera H., Nakata K., Uematsu K., Su C.-Y., Kawase T. (2016). Growth factor and pro-inflammatory cytokine contents in platelet-rich plasma (PRP), plasma rich in growth factors (PRGF), advanced platelet-rich fibrin (A-PRF), and concentrated growth factors (CGF). Int. J. Implant Dent..

[12] Wang X., Zhang Y., Choukroun J., Ghanaati S., Miron R.J. (2017). Effects of an injectable platelet-rich fibrin on osteoblast behavior and bone tissue formation in comparison to platelet-rich plasma. Platelets.

[13] Liu Y., Sun X., Yu J., Wang J., Zhai P., Chen S., Liu M., Zhou Y. (2019). Platelet-Rich Fibrin as a Bone Graft Material in Oral and Maxillofacial Bone Regeneration: Classification and Summary for Better Application. BioMed Res. Int..

[14] Rodella L.F., Favero G., Boninsegna R., Buffoli B., Labanca M., Scarì G., Sacco L., Batani T., Rezzani R. (2011). Growth factors, CD34 positive cells, and fibrin network analysis in concentrated growth factors fraction. Microsc. Res. Tech..

[15] Palermo A., Ferrante F., Stanca E., Damiano F., Gnoni A., Batani T., Carluccio M.A., Demitri C., Siculella L. (2019). Release of VEGF from dental implant surface (IML^®^ Implant) coated with Concentrated Growth Factors (CGF) and the Liquid Phase of CGF (LPCGF): In vitro results and future expectations. Appl. Sci..

[16] Wang L., Wan M., Li Z., Zhong N., Liang D., Ge L. (2019). A comparative study of the effects of concentrated growth factors in two different forms on osteogenesis in vitro. Mol. Med. Rep..

[17] Bianco P., Cao X., Frenette P.S., Mao J.J., Robey P.G., Simmons P.J., Wang C.-Y. (2013). The meaning, the sense and the significance: Translating the science of mesenchymal stem cells into medicine. Nat. Med..

[18] Abdallah B.M., Kassem M. (2007). Human mesenchymal stem cells: From basic biology to clinical applications. Gene Ther..

[19] Abdallah B.M., Ditzel N., Kassem M. (2008). Assessment of bone formation capacity using in vivo transplantation assays: Procedure and tissue analysis. Adv. Struct. Saf. Stud..

[20] Sacchetti B., Funari A., Michienzi S., Di Cesare S., Piersanti S., Saggio I., Tagliafico E., Ferrari S., Robey P.G., Riminucci M. (2007). Self-renewing osteoprogenitors in bone marrow sinusoids can organize a hematopoietic microenvironment. Cell.

[21] Li J., Huang Z., Li B., Zhang Z., Liu L. (2019). Mobilization of transplanted bone marrow mesenchymal stem cells by erythropoietin facilitates the reconstruction of segmental bone defect. Stem Cells Int..

[22] Toyama N., Tsuchiya S., Kamio H., Okabe K., Kuroda K., Okido M., Hibi H. (2020). The effect of macrophages on an atmospheric pressure plasma-treated titanium membrane with bone marrow stem cells in a model of guided bone regeneration. J. Mater. Sci. Mater. Med..

[23] Langenbach F., Handschel J. (2013). Effects of dexamethasone, ascorbic acid and β-glycerophosphate on the osteogenic differentiation of stem cells in vitro. Stem Cell Res. Ther..

[24] Logan N., Camman M., Williams G., Higgins C.A. (2018). Demethylation of ITGAV accelerates osteogenic differentiation in a blast-induced heterotopic ossification in vitro cell culture model. Bone.

[25] Hanna H., Mir L.M., Andre F.M. (2018). In vitro osteoblastic differentiation of mesenchymal stem cells generates cell layers with distinct properties. Stem Cell Res. Ther..

[26] Zhang X., Wang Y., Zhao H., Han X., Zhao T., Qu P., Li G., Wang W. (2020). Extracellular vesicle-encapsulated miR-22-3p from bone marrow mesenchymal stem cell promotes osteogenic differentiation via FTO inhibition. Stem Cell Res. Ther..

[27] Ullah I., Subbarao R.B., Rho G.J. (2015). Human mesenchymal stem cells-current trends and future prospective. Biosci. Rep..

[28] Gassling V., Açil Y., Springer I.N., Hubert N., Wiltfang J. (2009). Platelet-rich Plasma and Platelet-rich fibrin in human cell culture. Oral Surg. Oral Med. Oral Pathol. Oral Radiol. Endodontol..

[29] Fernandes G., Yang S. (2016). Application of platelet-rich plasma with stem cells in bone and periodontal tissue engineering. Bone Res..

[30] Hatakeyama I., Marukawa E., Takahashi Y., Omura K. (2013). Effects of platelet-poor plasma, platelet-rich plasma, and platelet-rich fibrin on healing of extraction sockets with buccal dehiscence in dogs. Tissue Eng. Part A.

[31] Pirraco R.P., Reis R.L., Marques A.P. (2012). Effect of monocytes/macrophages on the early osteogenic differentiation of hBMSCs. J. Tissue Eng. Regen. Med..

[32] Rutkovskiy A., Stensløkken K.-O., Vaage I.J. (2016). Osteoblast differentiation at a glance. Med. Sci. Monit. Basic Res..

[33] Komori T. (2009). Regulation of bone development and extracellular matrix protein genes by RUNX2. Cell Tissue Res..

[34] Jonason J., Xiao G., Zhang M., Xing L., Chen D. (2009). Post-translational regulation of Runx2 in bone and cartilage. J. Dent. Res..

[35] Shui C., Spelsberg T.C., Riggs B.L., Khosla S. (2003). Changes in Runx2/Cbfa1 expression and activity during osteoblastic differentiation of human bone marrow stromal cells. J. Bone Miner. Res..

[36] Hamidouche Z., Haÿ E., Vaudin P., Charbord P., Schüle R., Marie P.J., Fromigué O. (2008). FHL2 mediates dexamethasone-induced mesenchymal cell differentiation into osteoblasts by activating Wnt/β-catenin signaling-dependent Runx2 expression. FASEB J..

[37] Hong D., Chen H.-X., Xue Y., Li D.-M., Wan X.-C., Ge R., Li J.-C. (2009). Osteoblastogenic effects of dexamethasone through upregulation of TAZ expression in rat mesenchymal stem cells. J. Steroid Biochem. Mol. Biol..

[38] Zaidi S.K., Javed A., Pratap J., Schroeder T.M., Westendorf J.J., Lian J.B., Van Wijnen A.J., Stein G.S., Stein J.L. (2006). Alterations in intranuclear localization of Runx2 affect biological activity. J. Cell. Physiol..

[39] Myllyharju J. (2004). Collagens, modifying enzymes and their mutations in humans, flies and worms. Trends Genet..

[40] Wu M., Chen G., Li Y.-P. (2016). TGF-β and BMP signaling in osteoblast, skeletal development, and bone formation, homeostasis and disease. Bone Res..

[41] Xu J., Li Z.-H., Hou Y., Fang W. (2015). Potential mechanisms underlying the Runx2 induced osteogenesis of bone marrow mesenchymal stem cells. Am. J. Transl. Res..

[42] Yu X., Ren H., Shang Q., Shen G., Tang K., Yu F., Chen G., Zhang Z., Zhao W., Zhang P. (2020). Effects of concentrated growth factor on the proliferation, migration, and osteogenic differentiation of rat bone marrow mesenchymal stem cells: An in vitro study. Res. Sq..

[43] Chen X., Chen Y., Hou Y., Song P., Zhou M., Nie M., Liu X. (2019). Modulation of proliferation and differentiation of gingiva-derived mesenchymal stem cells by concentrated growth factors: Potential implications in tissue engineering for dental regeneration and repair. Int. J. Mol. Med..

[44] Zhang L., Ai H. (2019). Concentrated growth factor promotes proliferation, osteogenic differentiation, and angiogenic potential of rabbit periosteum-derived cells in vitro. J. Orthop. Surg. Res..

[45] Yu B., Wang Z. (2013). Effect of concentrated growth factors on beagle periodontal ligament stem cells in vitro. Mol. Med. Rep..

[46] Ma X., Ding L., Tang S., Li T., Pei J., Li Y. (2018). Effects of concentrated growth factors on proliferation and osteogenic differentiation in Beagle adipose-derived stem cells. Zhong Nan Da Xue Xue Bao Yi Xue Ban.

[47] Gersch R.P., Glahn J., Tecce M.G., Wilson A.J., Percec I. (2017). Platelet-rich plasma increases stem cells derived from fat cells growth and differentiation. Aesthetic Surg. J..

[48] Xu Y., Qiu J., Sun Q., Yan S., Wang W., Yang P., Song A. (2019). One-year results evaluating the effects of concentrated growth factors on the healing of intrabony defects treated with or without bone substitute in chronic periodontitis. Med. Sci. Monit..

[49] Jin R., Song G., Chai J., Gou X., Yuan G., Chen Z. (2018). Effects of concentrated growth factor on proliferation, migration, and differentiation of human dental pulp stem cells in vitro. J. Tissue Eng..

[50] Tabatabaei F.S., Aghamohammadi Z., Tayebi L. (2020). In vitro and in vivo effects of concentrated growth factor on cells and tissues. J. Biomed. Mater. Res. Part A.

[51] Lokwani B.V., Gupta D., Agrawal R.S., Mehta S., Nirmal N.J. (2020). The use of concentrated growth factor in dental implantology: A systematic review. J. Indian Prosthodont. Soc..

[52] Rochira A., Damiano F., Marsigliante S., Gnoni G.V., Siculella L. (2013). 3,5-Diiodo-l-thyronine induces SREBP-1 proteolytic cleavage block and apoptosis in human hepatoma (Hepg2) cells. Biochim. Biophys. Acta (BBA) Mol. Cell Biol. Lipids.

