# Supplementary material for: Concentrated Growth Factors (CGF) Induce Osteogenic Differentiation in Human Bone Marrow Stem Cells
Source: Biology (Basel). 2020 Oct 30;9(11):370. doi: 10.3390/biology9110370 (PMC7693660; doi:10.3390/biology9110370)
Supplement: Supplementary file 1 [file biology-09-00370-s001.pdf]

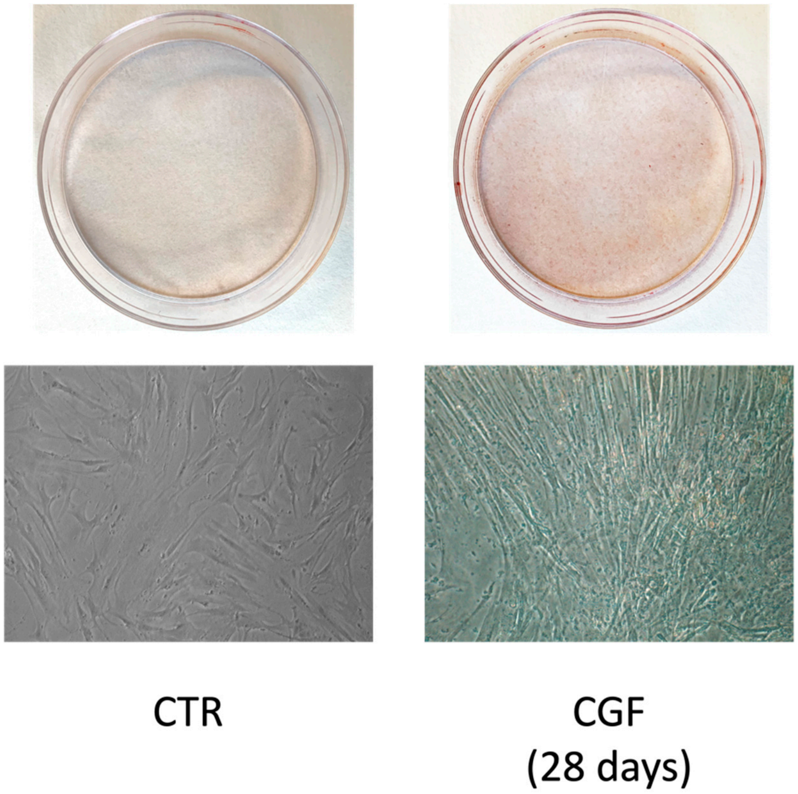


**Figure S1.** Alizarin Red staining in hBMSC cultured in MSC Basal Medium (BM) (Control, CTR), and BM + CGF (CGF 28 days), for 28 days (20x).
